# Experimental human-like model to assess the part of viable *Legionella* reaching the thoracic region after nebulization

**DOI:** 10.1371/journal.pone.0186042

**Published:** 2017-10-05

**Authors:** Jérémie Pourchez, Lara Leclerc, Françoise Girardot, Serge Riffard, Nathalie Prevot, Séverine Allegra

**Affiliations:** 1 University of Lyon, Ecole Nationale Supérieure des Mines - Saint-Etienne, CIS-EMSE, SAINBIOSE, INSERM U1059, Saint Etienne, France; 2 University of Lyon, UJM-Saint-Etienne, CNRS, EVS-ISTHME UMR 5600, Saint-Etienne, France; 3 Nuclear Medicine Department, University Hospital Saint-Etienne, Saint-Etienne, France; Public Health England, UNITED KINGDOM

## Abstract

The incidence of Legionnaires’ disease (LD) in European countries and the USA has been constantly increasing since 1998. Infection of humans occurs through aerosol inhalation. To bridge the existing gap between the concentration of *Legionella* in a water network and the deposition of bacteria within the thoracic region (assessment of the number of viable *Legionella*), we validated a model mimicking realistic exposure through the use of (i) recent technology for aerosol generation and (ii) a 3D replicate of the human upper respiratory tract. The model’s sensitivity was determined by monitoring the deposition of (i) aerosolized water and Tc^99m^ radio-aerosol as controls, and (ii) bioaerosols generated from both *Escherichia coli* and *Legionella pneumophila* sg 1 suspensions. The numbers of viable *Legionella* prior to and after nebulization were provided by culture, flow cytometry and qPCR. This study was designed to obtain more realistic data on aerosol inhalation (*vs*. animal experimentation) and deposition at the thoracic region in the context of LD. Upon nebulization, 40% and 48% of the initial *Legionella* inoculum was made of cultivable and non-cultivable cells, respectively; 0.7% of both populations reached the filter holder mimicking the thoracic region in this setup. These results are in agreement with experimental data based on quantitative microbial risk assessment methods and bring new methods that may be useful for preventing LD.

## Introduction

*Legionella* (gram-negative bacilli) are ubiquitous in natural and anthropogenic aquatic ecosystems. They are responsible for severe pneumonia that may be fatal in 30% of cases when considering nosocomial infections. *L*. *pneumophila* is, by far, the most frequent species associated with Legionnaires’ disease (LD). The incidence of LD in European countries per million population increased from 4.3 to 11.5 during 1998–2012 [1,2]. A recent publication on drinking water shows that *Legionella* spp. were responsible for 66% of outbreaks and 26% of illnesses in the USA between 2011–2012 [3]. Surveillance of *Legionella* is based on water sample analysis by a standardized culture assay (AFNOR T90-431 / ISO 11731) and/or standardized real-time PCR method (AFNOR T90-471 / ISO 12869). Our previous studies on *Legionella* reservoirs have shown that whatever the method employed, it is difficult to correlate the concentration of *Legionella* and the infectious risk probably due to the presence of viable but non-culturable *Legionella* (VBNC) in water samples, and to a lesser extent, the presence of PCR and culture inhibitors [4–8]. Indeed, at least 14 physiological forms of *L*. *pneumophila* have been described in the environment [9]. Among them, the presence of VBNC forms, such as those deriving from exponential phase forms (EPFs) and stationary phase forms (SPFs), in water samples contributes to the number of potentially pathogenic *Legionella* being underestimated. In a previous study, we have shown that VBNC *Legionella* deriving from these forms were infectious for macrophage-like cells [7].

Moreover, human infection occurs almost exclusively through aerosol inhalation. Aerosols drive *Legionella* to the lungs. It is well documented that the bacteria multiply within a reprogrammed *Legionella*-specific vacuole into alveolar macrophages and some other cells of the respiratory mucosa [10–12]. To date, very few studies aimed at evaluating the concentration of *Legionella* in aerosolized particles have been published. For those available, discrepant results do not favour their use in the field [13–16]. To assess the risk of LD infection, QMRA (Quantitative Microbial Risk Assessment) methods provide models for aerosol dispersion [17–21]. However, these risk models are based on infectious doses extrapolated to humans from animal inhalation [22–26], intraperitoneal injection or tracheal instillation studies [27,28].

To bridge the existing gap between the concentration of *Legionella* in water distribution systems and the LD infectious doses for humans, there is a need to propose an original human-like respiratory model coupled with an experimental setup of *Legionella* aerosol generation. In this study, we describe a model mimicking realistic conditions of exposure to *Legionella* through the use of the following:

1. A vibrating-mesh nebulization system, which is a recent technology for aerosol generation [29,30]. Such nebulizers mimic the dispersion of aerosols generated by various devices (showers, cooling towers) connected to water distribution systems (see S1 Fig).
2. A 3D printed replicate of the human upper respiratory tract (initially optimized from a plastinated nasal cast) previously developed [30–33] and connected to a filter mimicking the thoracic region.

This study is the first to account for both the physiological status of aerosolized *Legionella* and the inoculum size that may reach the lungs. Moreover, this article demonstrates the usefulness of our human-like respiratory model dedicated to *Legionella* aerosol deposition. Finally, new insights into aerosol dispersion, transport and human exposure to LD are provided.

## Results

### Development and validation of a respiratory model dedicated to *Legionella* aerosol deposition

#### Set-up description

**Fig 1:**
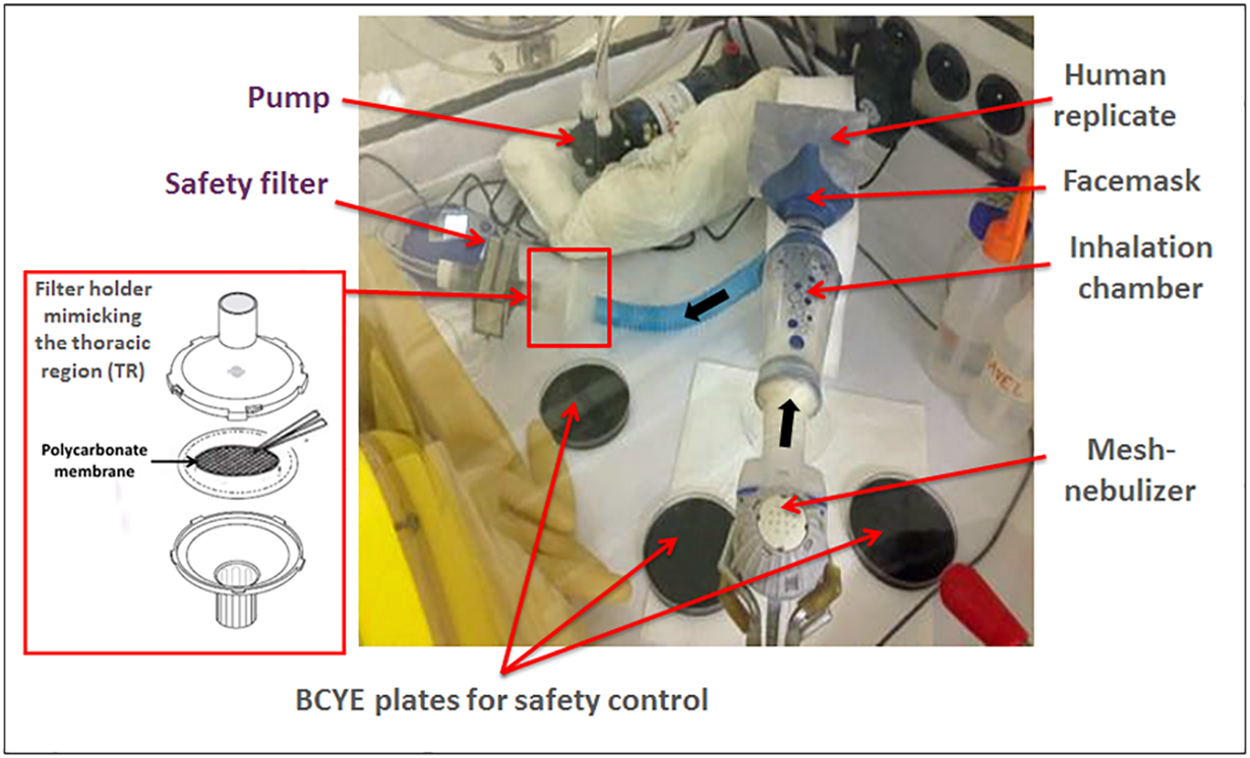
Human-like experimental model inside a glove box. This experimental setup allows the dispersion and collection of *Legionella* aerosols reaching the filter holder mimicking the thoracic region. Black arrows indicate the direction of airflow.

Our study has benefited from the newest technology available on the nebulizer market for aerosol generation, a vibrating-mesh nebulization [29,30]. As shown in Fig 1, the setup was linked to a pump simulating inspiration and allowing aerosol dispersion from the mesh-nebulizer between 0.6 and 0.9 mL.min^-1^. The human replicate was connected to the mesh-nebulizer through an inhalation chamber. The thoracic region (TR) was simulated by a filter holder (FH) with a polycarbonate membrane. The TR was connected to the human replicate through an artificial trachea. This anatomically realistic upper airway model maintains the natural curvature and volume of the upper airway and its original aerodynamic behaviour [31].

#### Safety control

First, the natural heterotrophic flora in the glove box and in the experimental room were measured. The first nebulization tests were carried out with sterile distilled water (aerosolized water) and suspensions of *Escherichia coli* strain 039 (*Ec 039*) in order to check the sealing of the respiratory model and potential contamination of the glove box and the experimental room.

After several rounds of optimization, no leaks were visually observed. Ten rounds of nebulization with sterile distilled water showed that only 0.35 ± 0.1 mL (i.e., 8.8%) of the water was lost.

Following the first experiments, only 147 out of 6.2x10^6^ CFU (i.e., 0.002%) of the aerosolized *Ec 039* were detected in the glove box. This value decreased to 24 out of 9x10^6^ CFU (i.e., 0.0003%). *Ec 039* was detected neither in the glove box airlock nor in the experimental room. The sealing of the experimental setup was also checked through aerosol nebulization of *Lp1 008-GFP* suspension with only 0.0001% of the initial aerosolized inoculum detected in the glove box. Therefore, we can assume that water leaks are negligible.

The weak presence of endogenous flora in the air of the experimental room and in the glove box (below the quantification limit of 4 bacteria according to NF EN ISO 8199 [34]) indicates that the manipulations were carried out in good conditions and without interference with the nebulization experiments.

#### The sensitivity of the model as determined by percentages of aerosol deposition within the experimental setup

**Fig 2:**
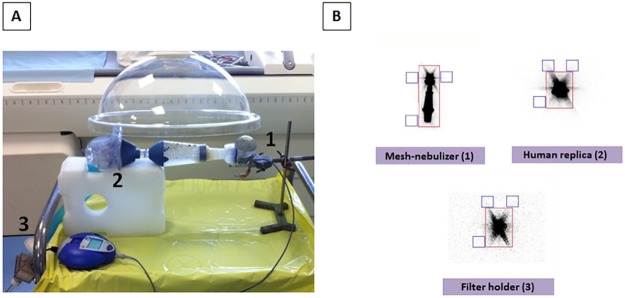
The sensitivity of the model as determined by 2D planar scintigraphy of Tc^99m^. (A) Aerosolization in the setup for 5 min. Mesh-nebulizer with inhalation chamber (1), Human replicate (2) and Filter holder (3). (B) 2D scintigraphic images recorded over a 2-min period for each element of the setup. Regions of interest (ROIs, boxed in red) were delimited on the images with correction of the background using a mean of 3 external ROIs (blue squares).

We monitored the deposition of aerosolized water and radiolabelled Tc^99m^ as aerosol controls and of bioaerosols (*Ec 039* and *Lp1 008-GFP*). The deposition of aerosols in the filter holder (FH) mimicking the thoracic region and of the non-aerosolized suspension in the mesh-nebulizer tank were assessed by weighing. The deposition of the aerosol radiolabelled with Tc^99m^ was visualized and quantified by 2D planar scintigraphy (Fig 2).

The weight percentage of water, the percentage of radioactivity for Tc^99m^ and the percentage of bacteria aerosolized and reaching the FH (Table 1) were calculated for all nebulization experiments (n = 40). For each experiment, 4.0 ± 0.3 mL (out of 5 mL in the nebulizer tank) was finally aerosolized. The time needed to disperse such a volume was 5.6 ± 1.2 min. Therefore, our model simulates total aerosol output between 0.6 and 0.9 mL.min^-1^. As shown in Table 1, no significant differences were observed between *Ec 039*, *Lp1 008-GFP* and aerosolized water, meaning that the presence of biological particles in the aerosols did not alter their pattern of deposition. The percentage reaching the FH, i.e., the thoracic region, varied from 2.4 to 7.4%. By contrast, the aerosol labelled radioactively using Tc^99m^ showed higher deposition in the thoracic region (9.4%) compared to the three other weighing methods (water aerosol and bioaerosol containing *Ec 039* or *Lp1 008*-GFP). In agreement with our previous studies [29–31], these results remained in the same 5–10% range of thoracic deposition. The difference between Tc^99m^ and the other markers is because the radiolabelling method is clearly more accurate and sensitive.

**Table 1:** Percentages of water, Tc^99m^ and aerosolized bacteria reaching the filter holder.

	Aerosols reaching FH (%) Mean ± SD (Min—Max)
Water (n = 10)	5.0 ± 1.3 (3.5–7.4)
*E*. *coli 039* (n = 10)	5.0 ± 1.1 (3.4–6.9)
*Lp1 008* (n = 20)	4.8 ± 1.4 (2.4–7.2)
Tc^99m^ (n = 3)	9.4 ± 4.0 (5–12.8)

The percentage reaching the filter holder (FH) was obtained by weighing the polycarbonate membrane for Water, *Ec 039* and *Lp1 008-GFP* assays. For Tc^99m^ radioactivity quantification, see Fig 2. n: number of samples.

### Quantification of total (viable and culturable (VC), VBNC and dead cells (DC)) bacteria

VC bacteria were assessed in the calibrated suspensions (CS) and in the aerosolized fraction reaching the FH using colony counts (by culture). The quantification of total bacteria was done by qPCR.

As shown in Table 2, the same percentage of VC reaching FH was observed when testing suspensions of *Ec 039* (n = 10) and *Lp1 008-GFP* (n = 20) (*p* = 0.07). The quantification of total bacteria shows no significant difference between the percentages of total *Ec 039* (1.81%) and *Lp1 008-GFP* (0.89%) reaching FH (*p* = 0.1).

**Table 2:** Quantification of viable and culturable (VC) cells by culture and total cells by qPCR.

		CS	FH	% at FH
**Culture** Number of VC (CFU) *p* = 0.07	*Ec 039* (n = 10)			
	Mean	9.8 x10^6^	7.6 x10^3^	0.14
	*SD*	*1*.*7 x10*^*7*^	*8*.*7 x10*^*3*^	*0*.*11*
	*min*	*7*.*3* x10^5^	*6*.*3* x10^2^	*0*.*02*
	*max*	*5*.*7* x10^7^	*2*.*9* x10^4^	*0*.*29*
	*Lp1 008*-GFP (n = 20)			
	Mean	2.1 x10^6^	3.1 x10^3^	0.23
	*SD*	*2*.*4 x10*^*6*^	*3*.*5* x10^3^	*0*.*17*
	*min*	*1*.*6* x10^4^	*6*.*5* x10^1^	*0*.*08*
	*max*	*7*.*3* x10^6^	*1*.*1* x10^4^	*0*.*71*
**qPCR** Number of bacteria (eq. CFU) *p* = 0.1	*Ec 039* (n = 10)			
	Mean	1.3 x10^9^	2.4 x10^6^	1.81
	*SD*	*2*.*3 x10*^*9*^	*3*.*9 x10*^*6*^	*2*.*19*
	*min*	*3*.*3* x10^6^	*8*.*4* x10^4^	*0*.*01*
	*max*	*9*.*2* x10^9^	*1*.*4* x10^7^	*6*.*35*
	*Lp1 008*-GFP (n = 20)			
	Mean	4.5 x10^7^	5.9 x10^4^	0.89
	*SD*	*9*.*6 x10*^*7*^	*1*.*8 x10*^*5*^	*1*.*92*
	*min*	*1*.*7* x10^4^	*8*.*2* x10^1^	*0*.*00*
	*max*	*5*.*0* x10^8^	*9*.*5* x10^5^	*8*.*88*

CS: calibrated suspension (quantification of bacteria in the 5 mL suspension deposited in the nebulizer tank). FH: Filter holder (quantification of bacteria reaching FH after nebulization). eq. CFU: equivalent CFU. n: number of samples. The percentage of bacteria reaching the FH has been calculated for each experiment (10 or 20 experiments for *Ec 039* and *Lp1 008-GFP* respectively). The percentages presented in the column “% at FH” (in grey) are the mean, the standard deviation (SD), the min and the max of the calculated percentage of bacteria reaching FH for each experiment.

**Fig 3:**
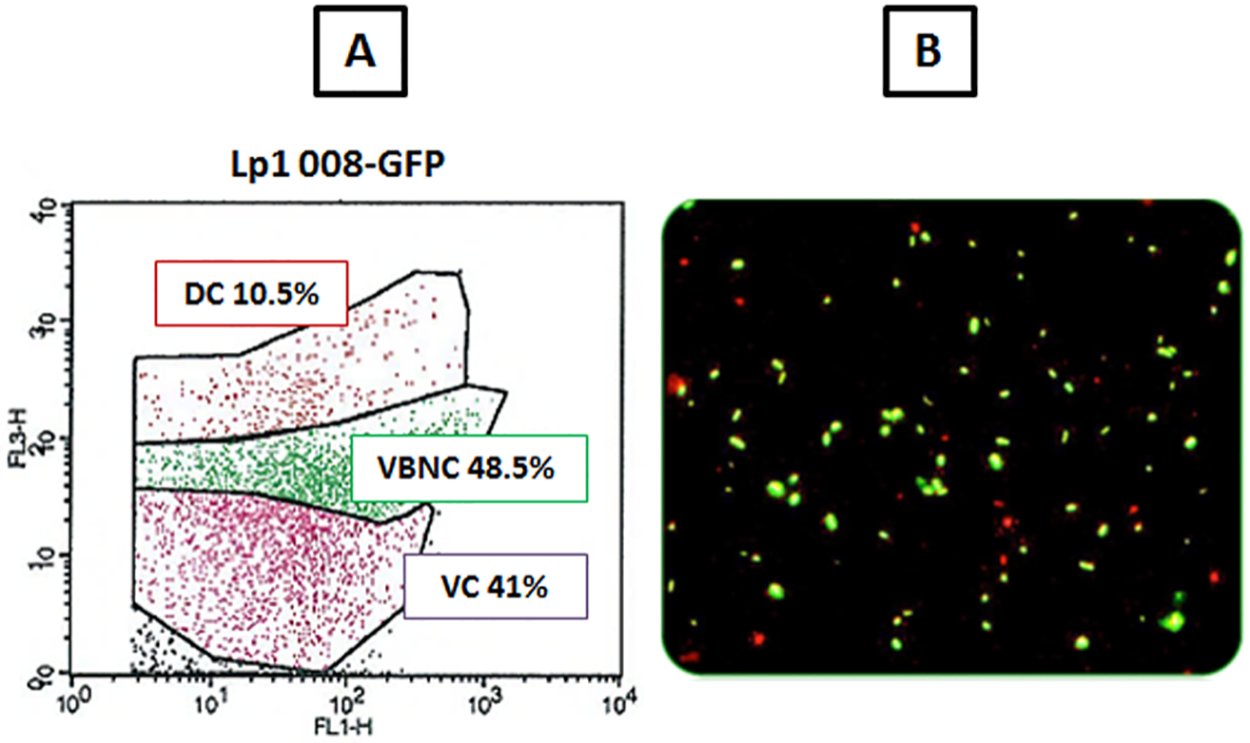
Determination of physiological state of *Legionella* aerosols in calibrated suspension before aerosolization. A. Representative analysis by FCA. B. Visual control for FCA by epifluorescence microscopy (400x). DC: Dead Cells (red bacteria by microscopy). VBNC: Viable But Not Culturable (double-labelled bacteria by microscopy). VC: Viable and Culturable (green bacteria by microscopy).

When using a flow cytometric assay (FCA), the detection limit of which is 1x10^3^ bacteria/mL, an *Lp1 008-GFP* calibrated suspension of 2x10^6^ CFU/mL was necessary considering that a mean of 0.89% of total *Legionella* cells can reach the FH (Table 2). Similar flow cytometric profiles (Fig 3A) were obtained for all the samples (n = 10). A decrease in the percentages of viable cells (VC and VBNC) upon nebulization is shown in Table 3.

**Table 3:** Effect of nebulization on the physiological forms of *Legionella*.

FCA (%) (*Lp1 008 -GFP*) (n = 10)	VC		VBNC		DC	
	CS	FH	CS	FH	CS	FH
**Mean**	**40.2**	**33.4**	**48.2**	**38.8**	**11.6**	**27.8**
Standard deviation	3.6	3.6	4.5	3.0	6.9	6.6

CS: calibrated suspension. FH: Filter holder (after nebulization). Viable and culturable cells (VC); viable but not culturable (VBNC) and dead cells (DC). n = number of samples.

Epifluorescence microscopy was used as a visual control for the FCA results (Fig 3B).

Knowing the mean percentage of each physiological form (by FCA) in the CS and in the FH and the total number of cells (eq. CFU by qPCR), we could estimate the number of other forms (Table 4, deduced from qPCR). In the same way, knowing the number of VC cells (CFU by culture), we could estimate the number of other forms (Table 4, deduced from culture). As qPCR is more sensitive (as qPCR can detect DNA and DNA fragments of dead cells), a higher percentage of *Legionella* reaching the FH (i.e., the thoracic region) is observed but there is no significant difference between the two methods (*p* = 0.07). Upon nebulization, 40% of the initial inoculum is made of cultivable cells (VC) and 48% of viable but not cultivable cells (VBNC). Only a small part of both VC and VBNC cells within the initial nebulized inoculum may reach the thoracic region mimicked by a filter holder (0.7% of both populations).

**Table 4:** Estimation of the number of physiological forms of *Legionella*.

*Lp1 008-GFP* (n = 20)	CS			FH			% at FH	
	FCA (%)	deduced from		FCA (%)	deduced from		deduced from	
		qPCR	Culture		qPCR	Culture	qPCR	Culture
Total cells	**100**	**4.5 x10**^**7**^	5.2 x10^6^	**100**	**5.9 x10**^**4**^	9.3 x10^3^	**0.89**	0.28
VC	**40.2**	1.8 x10^7^	**2.1 x10**^**6**^	**33.4**	2.0 x10^4^	**3.1 x10**^**3**^	0.74	**0.23**
VBNC	**48.2**	2.2 x10^7^	2.5 x10^6^	**38.8**	2.3 x10^4^	3.6 x10^3^	0.71	0.23
DC	**11.6**	5.2 x10^6^	6.1 x10^5^	**27.8**	1.6 x10^4^	2.6 x10^3^	2.12	0.68

qPCR results are expressed in CFU equivalents (see the Materials and methods section). Culture results are expressed in CFU. FCA results are in percentages. Bold numbers are experimental data. Other figures were deduced from these data. CS: calibrated suspensions. FH: Filter holder. n: number of samples.

### Extrapolation from French target values

The current French target values for *Legionella* control in water networks are alert value: 1x10^3^ *Legionella*/L and action value: 1x10^5^ *Legionella*/L. In this study, our purpose was to evaluate the number of *Legionella* that can reach the thoracic region if a concentration of *Legionella* equal to the French guideline values was present in the hot water distribution system. Unfortunately, considering that we could not aerosolize suspensions with bacterial concentrations lower than the detection limit of the FCA method, we extrapolated the numbers of viable *Legionella* reaching the FH if theoretical exposures of 1x10^3^ or 1x10^5^ *Legionella* per litre were considered. In our calculations, to mimic a shower, we considered an exposure of 20 minutes under a nebulization rate of 0.6 to 0.9 mL/min (Table 5).

**Table 5:** Numbers of viable (VC + VBNC) *Legionella* aerosolized and reaching FH (mimicking thoracic region) if extrapolated from French target values.

Theoretical concentration of *Legionella* in water network (CFU/L)	1.0 x10^3 (a)^	1.0 x10^5 (b)^
Number of *Legionella* aerosolized (CS—extrapolated from our model)	26–40	2640–3960
Number of *Legionella* reaching FH (extrapolated from our model)	**0.2–0.3**	**19–29**

(a) alert value (b) action value (37). Extrapolation was done for an exposure of 20 min at 0.6 to 0.9 mL/min.

Table 5 shows that a maximum of 0.3 and 29 CFU can reach the FH mimicking the thoracic region for the French alert and action values, respectively.

## Discussion

The proliferation of *Legionella* in sanitary hot water systems is controlled through the use of heat shock or chlorination treatment. Nevertheless, *Legionella* is currently the aetiologic agent most frequently associated with community water system outbreaks (65.6%) in the USA [3]. In Europe, the number of LD cases is mainly correlated with exposure to cooling towers (58%), water systems (26%) and pools (1%) [35].

*Legionella* control in water systems is associated with concentration thresholds (Colony Forming Units—CFU) that have been empirically defined. In the case of sanitary hot water and for the general population, the French regulation has set the alert value for *L*. *pneumophila* to 1x10^3^ CFU.L^-1^. For high-risk patients (such as those immunosuppressed), the regulation is not to exceed the detection limit of the culture method [36]. For cooling towers, the Ministry of Environment has defined two target values for *L*. *pneumophila* [37]. The first threshold (1x10^3^ CFU.L^-1^) is associated with the need to undertake measures to control *Legionella* proliferation (alert value); the second (1x10^5^ CFU.L^-1^) is correlated with an immediate shutdown of the facility (action value).

Because infection of humans occurs through aerosol inhalation, in this study, we describe a model that simulates exposure close to that under realistic conditions. Indeed, our model mimics (i) the way aerosols are produced by showers or cooling towers (nebulization) and (ii) a human upper respiratory tract [19,38]. Compared to jet nebulizers, vibrating-mesh nebulizers generate reproducible aerosols of respirable size (see S1 Fig) and there is a lower residual volume in the reservoir [39].

This study is the first to account for both the physiological status of aerosolized *Legionella* and the inoculum size that may reach the thoracic region. In a previous study on the characterization of aerosols containing *Legionella*, we showed that direct nebulization into a low-pressure cascade impactor (DLPI) had no impact on *Legionella* viability [29]. However, in the current study, because of mechanical constraints, the airborne transport from the nebulizer through the inhalation chamber, human head replicate and trachea killed a significant portion of the inoculum (Fig 3 and Table 3). It is not clear whether this phenomenon is due to the lack of mucosal layers within the upper respiratory tract replicate. Despite this, our setup is more realistic than any animal model [40], as we generate *Legionella* aerosols on a human head replicate. To date, to our knowledge, this model remains the most relevant for such purposes.

Extrapolating from our model (Table 5), an exposure of 20 min (with aerosol dispersion between 0.6 and 0.9 mL.min^-1^ from the calibrated suspension) with 1x10^5^ *Legionella* is likely to carry approximately 30 viable bacteria to the thoracic region. According to the animal infections experiments as well as those of Armstrong *et al*. on QMRA (Quantitative Microbial Risk Assessment) models, 30 bacteria are not sufficient to cause infection to people with a "normal" immune system. But, according to statistics available, most LD cases are related with immunosuppressed patients. Therefore, this French action value of 1x10^5^ *Legionella*/L (correlated with an immediate shutdown of the facilities), could be decreased for facilities that can generate aerosols in contact with immunosuppressed persons.

To assess the risk of LD infection, the QMRA method provides models for aerosol dispersion. Hines et al. [22] confirmed that a screening-level exposure assessment approach can yield applicable data to assess the contribution of water uses to potential *Legionella* exposure. However, QMRA methods are still hindered by the difficulty of properly assessing both the emission and exposure factors and the physiological role of the respiratory mucosa. Our model provides experimental data following nebulization as an exposure factor. However, the current model does not address deposition at the alveoli, which is the target organ for initiation of *Legionella* infection. In the same manner, some removal of aerosols is likely to occur between the upper and lower respiratory pathways. Further experiments using an *ex vivo* human-like model are in progress to investigate the physiological role of the respiratory mucosa in the *Legionella* infection process. In this model, the filter holder (FH) is replaced by porcine *ex vivo* lungs.

There is still a need to evaluate whether an exposure of 20 minutes during a shower is comparable (the number of aerosolized bacteria reaching the lungs) to an exposure using our model of nebulization.

## Materials and methods

### Experimental procedure

The calibrated suspensions were analysed before and after nebulization in the experimental model.

### Bacterial culture, suspensions preparation and characterization

An *Escherichia coli (Ec 039)* strain and a *Legionella pneumophila* serogroup 1 (*Lp1 008-GFP*) GFP-modified strain were used [41,42]. They were stored at -80°C in Cryobank tubes (Mast Diagnostic, France). After thawing, *Lp1 008-GFP* and *Ec 039* were plated, respectively, onto BCYE agar (Buffered Charcoal Yeast Extract, SR0110 C, Oxoid, France) and Luria Broth (LB) agar supplemented with chloramphenicol (Sigma Aldrich, France) at 8 mg/mL (for GFP plasmid selection) for 24 h *(Ec 039)* or 72 h *(Lp1 008-GFP)* at 37°C. They were then re-plated onto the same medium and incubated at 37°C for another 24 h *(Ec 039)* or for 3 days (*Lp1 008-GFP)*. These cultures were then used to achieve a 10-mL calibrated suspension (CS) in sterile normal saline (0.9% NaCl) water. The final concentrations tested were 2x10^6^ and 2x10^5^ CFU/mL for *Legionella* and 2x10^6^ CFU/mL for *E*. *coli*.

### Description of the experimental model

An original human experimental respiratory model mimicking the upper and lower respiratory systems was used in this study. The setup was composed of a human resin replicate corresponding to the entire nasal cavity (nasal fossae, frontal, ethmoid and maxillary sinuses) without the oral cavity. This replicate was obtained after 3D reconstruction of a human head after a precise computerized tomography scan (CT-scan) as described in previous studies [31,33]. For this study, the replicate was manufactured in transparent water-resistant and non-porous resin. Endoscopy and qualitative CT-scan observations were performed on the replicate in order to assess anatomical reproducibility with a human plastinated known model [32]. Precise measurements were compared in critical anatomical regions: ostia and maxillary sinuses.

As shown in Fig 1, the human replicate was linked to a pump (model 420–2902–230 VAC, 50 Hz, 0.6 Amp, 1 PH, 0.02 HP, Thermo Scientific, France) simulating inhalation and allowing aerosol dispersion between 0.6 and 0.9 mL.min^-1^ of the calibrated suspensions. To avoid any bacterial contamination of the pump, an environmental filter (PALL filter BB50TE, PALL Medical, France) was placed after the thoracic filters. The thoracic region (TR) was simulated by a filter holder (Filter kit and filter PAD PARI, PulmoMed, France) with a polycarbonate membrane (Membrane filters Nuclepore^™^ track etched, Whatman, GE Healthcare, France) that allowed optimal bacterial recovery (according the standard procedures (AFNOR T90-431 / ISO 11731). Five millilitres of the calibrated suspension was nebulized in the respiratory model through an inhalation chamber (ID Tandem). The mesh-nebulizer (eRapid^®^ Nebulizer System by PARI, Cystic Fibrosis Services, France) used to aerosolize the bacterial suspension is an e-base nebulizer linked to a small compressor that delivered aerosols with a vibrating membrane technology (see S1 Fig). For safety reasons, the model was placed in a glove box (815-PGB “LA PETITE” GLOVE BOX, Fisher Scientific, France).

### Retrieving bacterial aerosols at thoracic region for subsequent analysis

To quantify the percentage of bacteria reaching the filter holder (the percentage of bacteria quantified at the target zone in relation to the inoculum concentration introduced into the nebulizer reservoir), the polycarbonate membrane (Fig 1) was weighed (1 g = 1 mL of water) before and after the nebulization. The membrane was placed in 5 mL of BYE (Buffered Yeast Extract, SR0110 C, Oxoid, France) or LB. After agitation for 30 s the membrane was scraped using an automatic pipette tip. One millilitre was plated twice on adapted culture medium to count viable and culturable (VC) bacteria; 1 mL was placed in a 1.5-mL tube for DNA extraction and qPCR analysis. For *Legionella* only, 1 mL was placed in a flow cytometry tube for flow cytometry (FCA) and microscopic analyses.

The same procedure was applied to the residual volumes of suspension remaining in the full setup: the volume not nebulized (staying in the nebulizer tank after nebulization) and the volumes collected from the human replicate, inhalation chamber and artificial trachea.

### Validation of model safety and sensitivity

After several rounds of experiments to optimize the sealing of the entire model, the lack of leaks in the experimental setup was checked by applying 10 rounds of nebulization with sterile distilled water. Leaks were assessed by visual control and weighing by comparing the volume of water aerosolized from the mesh-nebulizer with the residual volume of water remaining in the full setup after nebulization. The percentage of water is the weight percentage of the water reaching the target zone in relation to the volume introduced into the reservoir of the nebulizer.

To evaluate potential contamination of the glove box and the experimental room, a second round of nebulization (n = 10) was carried out with *Ec 039*. During these experiments opened bacterial agar dishes were distributed inside and outside of the glove box (Fig 2). This procedure was systematically applied during subsequent nebulization assays.

The dispersion of aerosols in the entire setup was assessed using Technetium 99m (Tc^99m^). Briefly, 4 mL of the radioactive suspension was aerosolized in the setup for 5 min (Fig 3). Radioactivity was measured over a 2-min period. Images of the setup were acquired by 2D planar scintigraphy with a planar gamma camera (resolution 128 × 128) using a single detector equipped with a low-energy, high-resolution collimator: E-cam camera (Siemens, Germany; 397 mm × 500 mm collimator) [43]. A region of interest (ROI) was delimited on the images with correction of the background using a mean of 3 external ROIs (Fig 3B). All calculations accounted for the background radiation and physical decay of radioactivity. The results were expressed in terms of the activity loaded into the nebulizer. These data allowed precise determination of the amount of radioactivity reaching the filter holder and the different regions of the setup. The results are expressed as the percentage of Tc^99m^, the percentage of the radioactive activity (thus Becquerel’s) reaching the target zone with respect to the radioactivity introduced into the reservoir of the nebulizer.

### qPCR

Quantification was performed using GFP mut2 sequence [44] expression by *Ec 039* and *Lp1 008-GFP*. The forward and reverse primers were, respectively, 5’- AGAGTGCCATGCCCGAAGGT -3’ and 5’- AAGGACAGGGCCATCGCCAA -3’. Plasmid DNA was extracted from all samples and a standard curve was made with the NuCleoSpin Plasmid kit (Macherey-Nagel, France) following the manufacturer’s instructions. qPCR was carried out on an ABI Prism 7500 automate (Applied Biosystems, France), using the 2X Power SYBR^®^ Green PCR Master Mix (Life Technologies, France) as follows: initial denaturation for 15 min and a two-step cycle consisting of 15 s denaturation, 1 min annealing and elongation at 60°C. At the end of each elongation step, the fluorescence of the incorporated SYBR Green dye was measured. At the end of 45 cycles of amplification, a melting curve program was incorporated to check for any primer dimers or other non-specific amplification. Each sample was run in duplicate. To express results in CFU equivalents, a standard curve was done with *Lp1 008-GFP* suspensions ranging from 2x10^7^ to 2x10^2^ CFU/mL. The results were analysed using Sequence Detection Software version 1.4 (ABI 7500 System Software, Applied Biosystems, France).

### Flow cytometric assay (FCA)

As previously described [5–7], FCA profiles of samples were obtained by using a combination of GFP green fluorescence (viable cells expressing GFP) and propidium iodide (PI) red fluorescence for cells with damaged membranes. Flow cytometric measurements were performed using a BD FACSCalibur instrument (Becton Dickinson Biosciences) equipped with an air-cooled argon laser (488-nm emission; 20 mW). The green fluorescent emission from GFP was collected in the FL1 channel (500 to 560 nm), and the red fluorescence from PI was collected in the FL3 channel (670 nm). A threshold was applied to the FL1 channel to eliminate background signals. Analyses were performed at a low-flow-rate setting. The results were analysed with Cell Quest Pro software (Becton Dickinson Biosciences).

### Epifluorescence microscopy

FCA samples (20 μL) were observed on a Nikon Eclipse Ti-S microscope equipped with a Nikon Digital Sight camera at a magnification of 400. The acquisition and image processing were done through Nis-D Element 3.0 software (Nikon).

### Statistics

The results are presented as the mean ± SD (standard deviation). We determined the statistical significance of differences between groups using Student’s *t*-test or ANOVA analyses. The value of *p* was considered significant for *p*<0.05. The statistical analysis was performed using StatView software version 5.0 (SAS Institute Inc., USA).

## Supporting information

S1 Fig Cumulative distribution of airborne droplets characterized by a low-pressure impactor (DLPI). *Legionella* nebulization and quantification by qPCR (expressed in % of genetic units reaching each stage of the DLPI, i.e., in % of total airborne *Legionella*). Particles greater than 10 μm were not plotted.
(DOCX)

S1 File Experimental procedure. VC: viable and culturable cells. VBNC: viable but not culturable cells. DC: dead cells. n: number of samples.
(TIF)
