# Supplementary material for: Experimental human-like model to assess the part of viable Legionella reaching the thoracic region after nebulization
Source: PLoS One. 2017 Oct 5;12(10):e0186042. doi: 10.1371/journal.pone.0186042 (PMC5628919; doi:10.1371/journal.pone.0186042)
Supplement: S1 Fig — Legionella nebulization and quantification by qPCR (expressed in % of genetic units reaching each stage of the DLPI, i.e., in % of total airborne Legionella). Particles greater than 10 μm were not plotted. (DOCX) [file pone.0186042.s001.docx]

**Supporting information.**

*Low pressure cascade impactor set-up*

The airborne particle size-distribution was assessed using a 13-stage cascade low pressure impactor (DLPI, Dekati, Finland). Operating at an airflow of 10 L.min^−1^, particles are impacted depending on their inertia-related aerodynamic diameter in one of the 13 size fractions stages. The size classification in DLPI is made from 30 nm up to 10 µm with evenly distributed impactor stages. In each size fraction the particles are collected on collection substrates to obtain gravimetric size distribution of the particles. Especially, the Median Aerodynamic Diameter (MAD) was calculated. The MAD was defined as the median of the distribution of airborne Legionella (expressed in % of GU) with respect to the aerodynamic diameter.

Median Aerodynamic Diameter (MAD) and Geometric Standard Deviation (GSD) of the particle size distribution, measured for *Legionella* nebulization with spacer device are respectively 1.60 ± 0.80 μm and 3.2 ± 0.5.


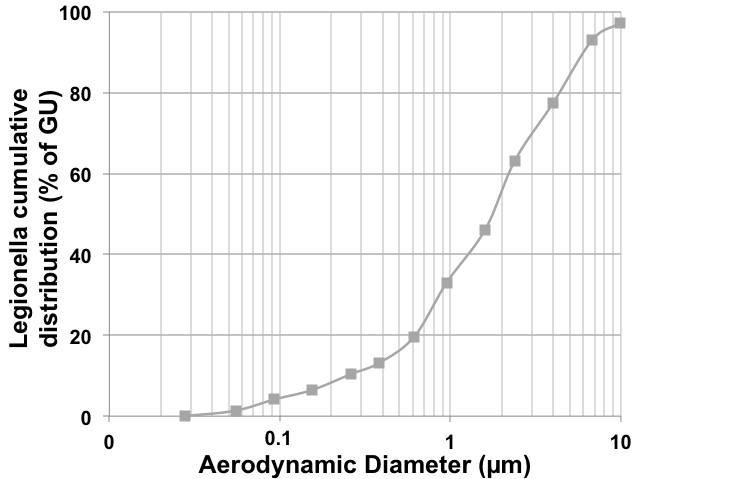


**S1 supporting information**: Cumulative distribution of airborne droplets characterized by low pressure impactor (DLPI). *Legionella* nebulization and quantification by qPCR (expressed in % of genetic unit reaching each stage of the DLPI, *i.e.* in % of total airborne *Legionella*). Particles greater than 10 µm were not plotted.
