# Supplementary figures and images for: Experimental human-like model to assess the part of viable Legionella reaching the thoracic region after nebulization
Source: PLoS One. 2017 Oct 5;12(10):e0186042. doi: 10.1371/journal.pone.0186042 (PMC5628919; doi:10.1371/journal.pone.0186042)

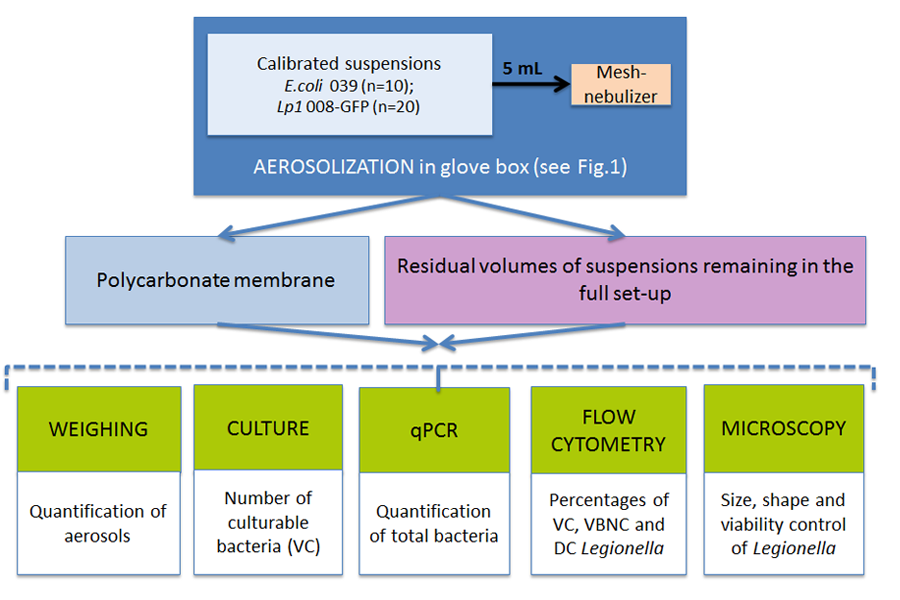

Supplement: S1 File — VC: viable and culturable cells. VBNC: viable but not culturable cells. DC: dead cells. n: number of samples. (TIF) [file pone.0186042.s002.tif]
